# Chest CT in patients after lung transplantation: A retrospective analysis to evaluate impact on image quality and radiation dose using spectral filtration tin-filtered imaging

**DOI:** 10.1371/journal.pone.0228376

**Published:** 2020-02-05

**Authors:** Alexander Wressnegger, Helmut Prosch, Bernhard Moser, Walter Klepetko, Peter Jaksch, Christopher Lambers, Konrad Hoetzenecker, Christian Schestak, Albert De Bettignies, Lucian Beer, Georg Apfaltrer, Helmut Ringl, Paul Apfaltrer

**Affiliations:** 1 Department of Biomedical Imaging and Image-guided Therapy, Medical University of Vienna, Vienna, Austria; 2 Division of Surgery, Department of Thoracic Surgery, Medical University Vienna, Vienna, Austria; 3 Division of Pediatric Radiology, Department of Radiology, Medical University of Graz, Graz, Austria; 4 Department of Neuroradiology, University Medical Center Mannheim, Medical Faculty Mannheim, University of Heidelberg, Mannheim, Germany; University of Oklahoma, UNITED STATES

## Abstract

**Objectives:**

The purpose of this study was to investigate the impact of a 150kV spectral filtration chest imaging protocol (Sn150kVp) combined with advanced modeled iterative reconstruction (ADMIRE) on radiation dose and image quality in patients after lung-transplantation.

**Methods:**

This study included 102 patients who had unenhanced chest-CT examinations available on both, a second-generation dual-source CT (DSCT) using standard protocol (100kVp, filtered-back-projection) and, on a third-generation DSCT using Sn150kVp protocol with ADMIRE. Signal-to-noise-ratio (SNR) was measured in 6 standardized regions. A 5-point Likert scale was used to evaluate subjective image quality. Radiation metrics were compared.

**Results:**

The mean time interval between the two acquisitions was 1.1±0.7 years. Mean-volume-CT-dose-index, dose-length-product and effective dose were significantly lower for Sn150kVp protocol (2.1±0.5mGy;72.6±16.9mGy*cm;1.3±0.3mSv) compared to 100kVp protocol (6.2±1.8mGy;203.6±55.6mGy*cm;3.7±1.0mSv) (p<0.001), equaling a 65% dose reduction. All studies were considered of diagnostic quality. SNR measured in lung tissue, air inside trachea, vertebral body and air outside the body was significantly higher in 100kVp protocol compared to Sn150kVp protocol (12.5±2.7vs.9.6±1.5;17.4±3.6vs.11.8±1.8;0.7±0.3vs.0.4±0.2;25.2±6.9vs.14.9±3.3;p<0.001). SNR measured in muscle tissue was significantly higher in Sn150kVp protocol (3.2±0.9vs.2.6±1.0;p<0.001). For SNR measured in descending aorta there was a trend towards higher values for Sn150kVp protocol (2.8±0.6 vs. 2.7±0.9;p = 0.3). Overall SNR was significantly higher in 100kVp protocol (5.0±4.0vs.4.0±4.0;p<0.001). On subjective analysis both protocols achieved a median Likert rating of 1 (25th-75th-percentile:1–1;p = 0.122). Interobserver agreement was good (intraclass correlation coefficient = 0.73).

**Conclusions:**

Combined use of 150kVp tin-filtered chest CT protocol with ADMIRE allows for significant dose reduction while maintaining highly diagnostic image quality in the follow up after lung transplantation when compared to a standard chest CT protocol using filtered back projection.

## Introduction

Patients after lung transplantation need to undergo several control chest CT examinations, which can lead to radiation dose escalation [1]. This patient cohort is especially prone to the possible risk of radiation-induced cancer due to the aggressive immunosuppressive therapy they need [2, 3]. Compared to the general population these patients have a 60-fold higher cancer risk not taking in account risks due to iatrogenic radiation exposure [4].

In recent years several important steps have been made to substantially reduce patients’ radiation dose in CT examinations. These include the use of iterative reconstruction (IR) algorithms instead of traditionally used filtered back projection (FBP), noise reduction filters, automated exposure control software, as well as modulation of tube current [3, 5–7].

In CT examinations without contrast media low-energy photons contribute little to nothing to image formation, because they get absorbed more than photons from the high-energy spectrum and due not reach the detector [7–9]. To increase the mean photon-energy range of examinations third-generation dual-source CTs are equipped with a 0.6 mm thick tin (Sn) filter in front of both X-ray tubes, that primarily absorbs photons of the low-energy range, a technique called spectral filtration [7].

IR is an image reconstruction algorithm introduced to improve image quality, enhance image resolution and lower image noise [10]. It achieves substantial radiation dose reduction, while at the same time, maintaining high diagnostic image quality when compared to FBP. This is not only true for the detection of pulmonary nodules, but also for the evaluation of tiny anatomical structures and lesions such as in diffuse lung disease [3, 11–14].

Recent studies showed that the use of spectral filtration with comparable high tube current protocols like 100kVp or 120kVp is dose efficient while maintaining high diagnostic image quality, even more so when combined with IR [8, 14–18]. Until now high kV spectral filtration imaging was only used for imaging of the parasinus region [19], however its effects on imaging of the lung parenchyma has not been investigated yet.

Consequently, the purpose of this study was to investigate the impact of a novel 150kV spectral filtration chest imaging protocol (Sn150kVp) combined with advanced modeled iterative reconstruction algorithms on radiation dose and image quality in the follow up of patients after lung-transplantation.

## Methods

### Subject population

The requirement for informed consent for this retrospective study got waived by our institutional review board (Ethics Committee of the Medical University of Vienna approval number: 1982). Between September 2016 and January 2017, 145 patients (74 male, mean age 51±14.7 years) were examined on a third-generation dual source CT (DSCT) using a spectral filtration unenhanced chest CT protocol (Sn150kVp) as a routine follow-up after lung-transplantation. 102 (70.3%) of these patients had also undergone a chest CT on a second-generation DSCT system previously using conventional 100kVp chest CT protocol and were consequently included in this study. No further inclusion or exclusion criteria were applied.

### CT technique

CT examinations were made on a third-generation 2 × 192-section dual-source CT system (SOMATOM Force; Siemens Healthineers) with a novel high kV spectral filtration chest protocol, 150kVp tube voltage, 96 mAs reference tube current, 0.25 s rotation time, pitch 1.2, 192 × 0.6 mm detector collimation, including the use of a 0.6 mm Sn filter placed directly after the X-ray source. Image reconstruction was performed using advanced modeled iterative reconstruction (ADMIRE) software (Siemens Healthineers) at strength level 3. ADMIRE offers five different strength levels, ranging from 1 (low) to 5 (high), a setting that primarily affects the level of noise reduction the algorithm aims at. Strength level 3 is offered as the default value by the instructor and showed to offer optimal results in both, image quality via noise reduction and radiation dose reduction, and was previously used in other studies investigating the effect of spectral filtration imaging in chest CT [8, 17] and represents the standard setting at our institution in this respect. The comparative examinations from the same patients were acquired on a second-generation DSCT (SOMATOM Definition Flash, Siemens Healthineers). The scanning parameters for these patients were: 100kVp tube voltage, 96 mAs reference tube current, 0.28 s rotation time, pitch 1.0, 128 × 0.6 mm detector collimation, no Sn filter. Automatic tube current modulation for effective mAs (CARE Dose4D, Siemens Healthineers) was used in all patients. Image reconstruction was performed using FBP. For detailed CT parameters see Table 1.

**Table 1:** CT parameters.

	3^rd^ generation CT	2^nd^ generation CT
**Scanner**	SOMATOM Force	SOMATOM Definition Flash
**kV**	Sn/150	100
**Ref mAs**	96	96
**Rotation Time (s)**	0.25	0.28
**Section thickness (mm)**	0.6	0.6
**Detector rows**	192	128
**Pitch**	1.2	1.0
**Image reconstruction**	ADMIRE	FBP

### Radiation dose estimates

Volume CT dose index (CTDIvol) values were documented in the dose report of the CT system for each CT study. Dose-length product (DLP), also given by the CT system, was used to estimate the individual radiation dose. To calculate effective dose (ED), a conversion factor was used according to the European Guidelines for Multislice Computed Tomography (dose conversion coefficients for the chest in mSv mGy^−1^ cm^−1^: 0.018 [20].

### Objective and subjective image quality

All images were evaluated on a dedicated image processing workstation (syngo MMWP VE 36A, Siemens Healthineers). Both objective and subjective image quality was assessed in a randomized fashion by two blinded observers (PA, experience in thoracic CT: 9 years; AW, experience in thoracic CT: 3 years).

**Fig 1:**
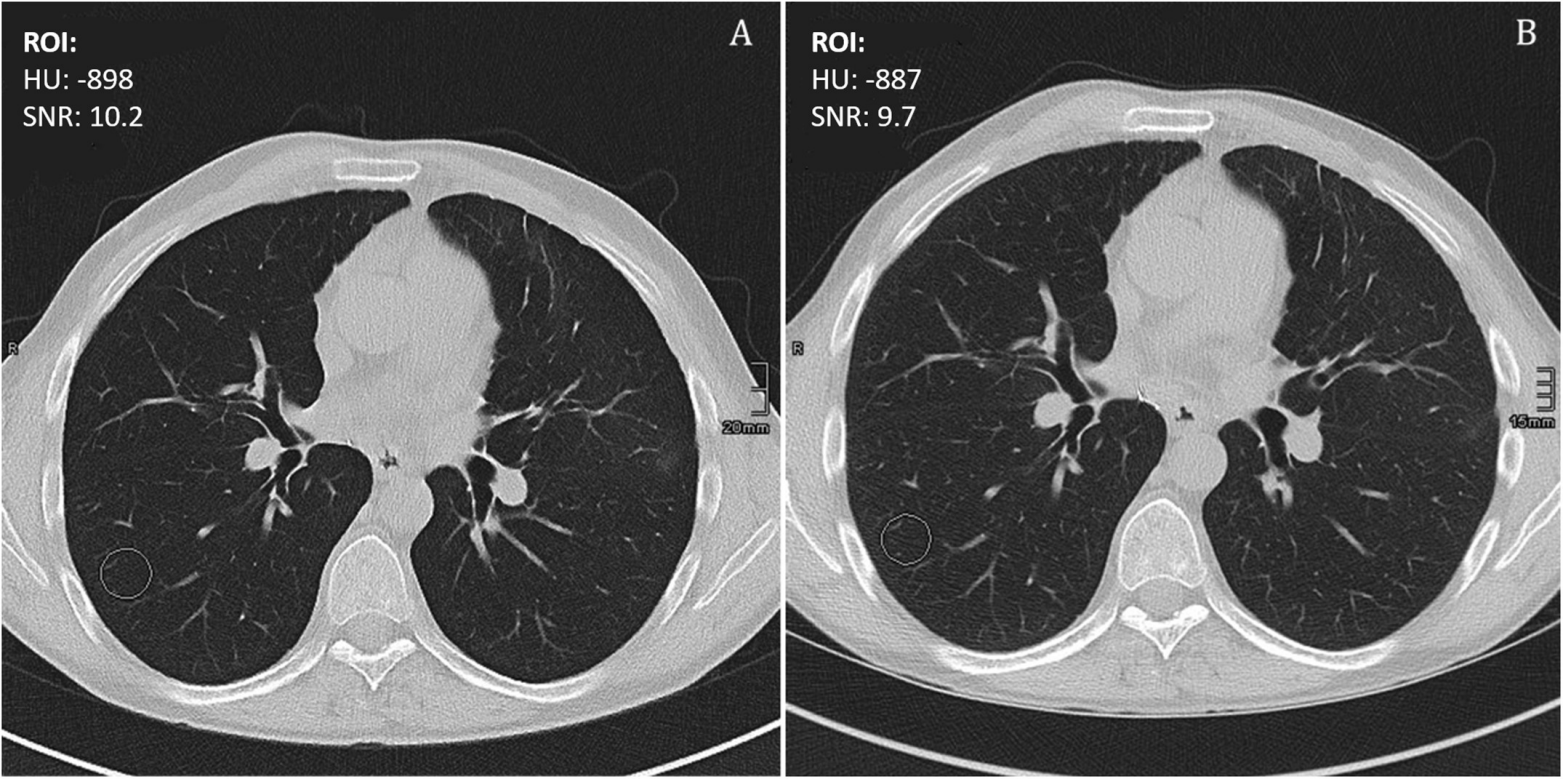
Quantitative image quality assessment. Example for the assessment of quantitative image quality of lung parenchyma by positioning a region of interest (ROI) and measuring Hounsfield units (HU) and image noise (standard deviation of the attenuation value). A) 3rd generation Sn150kVp protocol. B) 2nd generation 100kVp protocol.

To assessment quantitative image quality six different regions of interest (ROIs) were placed in identical positions in all examinations. These positions were in the lung tissue (avoiding large blood vessels and bronchi), the air within the trachea (directly above carina), the bone (vertebral body), and the background air (ie, outside the body) on the lung convolution kernel (Bl57) reconstruction, and the descending aorta (directly below level of left subclavian artery), and the muscle tissue (autochthonous back musculature) on the soft tissue convolution kernel (Br36). In all of these ROIs attenuation values in Hounsfield units (HU) and the standard deviation of the attenuation value as a parameter for image noise were measured. Afterwards the mean attenuation was divided by the noise of the identical ROI to calculate the signal-to-noise ratio (SNR). Examples of these measurements are presented in Fig 1.

To assess subjective image quality all images were anonymized and aggregated in folders in a random way to be evaluated by the two readers. Evaluation took place on both, a lung window setting (window level, -500; window width, 1,400) and a soft tissue window setting (window level, 40; window width, 350). Both radiologists evaluated normal lung structures (major fissures, small vessels and bronchi ≅2 mm in diameter), as well as main diagnoses and elementary signs after lung transplantation based on a five-point Likert scale [3, 5, 8] defined in Table 2 (reference adapted from *Haubenreisser et al* [8].

**Table 2:** Likert scale for subjective image quality.

Points	Definition
1 (excellent)	anatomical structures and pathological findings visible at 100%
2 (good)	anatomical structures and pathological findings visible at > 75%
3 (fair)	anatomical structures and pathological findings visible between 25% and 75%
4 (poor)	anatomical structures and pathological findings visible < 25%
5 (unacceptable)	Landmarks not visible at all

Assessment of complications and pathological signs after lung transplantation were assessed on the follow up Sn150kVp CT by one reader (PA, experience in thoracic CT: 9 years) adapted from Debray et al [21].

### Statistical analysis

Statistical analysis was performed using SPSS 25 (IBM, Armonk, NY, USA). For continuous variables results were expressed as means and standard deviations. For categorical variables frequencies and percentages were used. Continuous variables were compared using the independent t-test in case of normally distributed data. For non-normally distributed data the Mann–Whitney U test was used. Anteroposterior (AP) and lateral (LAT) chest diameter was compared on an intraindividual basis using paired t-test. Ordinal variables (image quality) are presented as median with 25^th^-75^th^ percentiles. To determine interobserver agreement for image quality, the intraclass correlation coefficient (ICC) was calculated. Substantial agreement was defined as ICC values of 0.61 to 0.80, excellent agreement as values of 0.81 to 1.00 [22]. P-values <0.05 were considered statistically significant.

## Results

### Patient characteristics

Underlying diseases that made lung transplantation necessary are summarized in Table 3. Thirty-three patients (32.3%) suffered from chronic obstructive pulmonary disease (COPD), 23 patients (22.5%) had cystic fibrosis, 22 interstitial lung disease with pulmonary fibrosis (21.6%), 12 patients (11.8%) had pulmonary arterial hypertension (PAH). Twelve patients (11.8%) suffered from other diseases such as alpha 1-antitrypsin deficiency and graft vs. host disease.

**Table 3:** Indications for lung transplantation.

n (%)	
33 (32.3%)	chronic obstructive pulmonary disease (COPD)
23 (22.5%)	cystic fibrosis
22 (21.6%)	interstitial lung disease with pulmonary fibrosis
12 (11.8%)	pulmonary arterial hypertension (PAH)
12 (11.8%)	others

Of 102 patients examined 94 (92.2%) had undergone double lung transplantation (DLUTX), whereas 8 (7.8%) were treated with single lung transplantation (LUTX). The main diagnoses observed was pleural effusion (11 patients; 10.8%), whereas the most observed elementary signs were consolidations (14 patients; 13.7%), bronchiectasis and centrilobular micronodules (both in 15 patients; 14.7%). Suspected fungal infection was found in one patient. Table 4 gives detailed information about main diagnoses and elementary signs observed. The mean follow-up period between lung transplantation and CT examination was 5.8±3.8 years in the Sn150kVp chest CT protocol group and 4.7±3.8 years in the standard protocol chest CT group (p = 0.039). AP and lateral chest diameters did not differ significantly between the two examination timepoints. Mean AP chest diameter was 225.4 mm in the Sn150kVp protocol vs. 224.1 mm in the 100kVp protocol (p = 0.236), mean LAT chest diameter was 347.9 mm vs. 348.7 mm (p = 0.719).

**Table 4:** Main diagnoses and elementary signs.

**Main diagnoses**	Fungal infection, n = 1
	Pneumothorax, n = 0
	Pleural effusion, n = 11
	Bronchial stenosis, n = 4
**Elementary signs**	Consolidations, n = 14
	Ground-glass opacities, n = 9
	Centrilobular micronodules, n = 15
	Nodules with halo, n = 1
	Nodules >1 cm, n = 0
	Reticulations, n = 7
	Bronchiectasis, n = 15

### Radiation dose

Mean CTDIvol and mean DLP was 2.1±0.5mGy and 72.6±16.9mGy*cm at Sn150kVp chest protocol versus 6.2±1.8mGy and 203.6±55.6mGy*cm at conventional chest CT protocol (p<0.001). Correspondingly, the mean effective dose was 1.3±0.3mSv at Sn150kVp chest protocol versus 3.7±1.0mSv at conventional chest CT protocol (p<0.001). This equals a 65% reduction in radiation dose for patient’s follow up CT after lung transplantation when compared to a standard chest CT protocol using FBP.

### Objective image quality

SNR measured in lung tissue, air inside trachea, vertebral body and air outside the body was significantly higher in conventional chest CT protocol compared to the Sn150kVp chest CT protocol (12.5±2.7 vs. 9.6±1.5, 17.4±3.6 vs. 11.8±1.8, 0.7±0.3 vs. 0.4±0.2, 25.2±6.9 vs. 14.9±3.3; all p<0.001). SNR measured in muscle tissue was significantly higher in the Sn150kVp chest CT protocol compared to the conventional chest CT protocol (3.2±0.9 vs. 2.6±1.0; p<0.001). For SNR measured in descending aorta there was a trend towards higher values for the Sn150kVp chest protocol compared to the conventional chest CT protocol (2.8±0.6 vs. 2.7±0.9), which was not statistically significant (p = 0.3). Overall SNR was also significantly higher in the conventional chest CT protocol (5.0±4.0 vs. 4.0±4.0; p = 0.001). For detailed results see Table 5.

**Table 5:** Objective image quality.

Region	Signal-to-noise ratio (SNR)		p-value
	3^rd^ generation CT	2^nd^ generation CT	
Lung tissue	9.6±1.5	12.5±2.7	<0.001
Air inside trachea	11.8±1.8	17.4±3.6	<0.001
Vertebral body	0.4±0.2	0.7±0.3	<0.001
Background air	14.9±3.3	25.2±6.9	<0.001
Descending aorta	2.8±0.6	2.7±0.9	0.3
Muscle tissue	3.2±0.9	2.6±1.0	<0.001
Mean	4.0±4.0	5.0±4.0	0.001

### Subjective image quality

**Fig 2:**
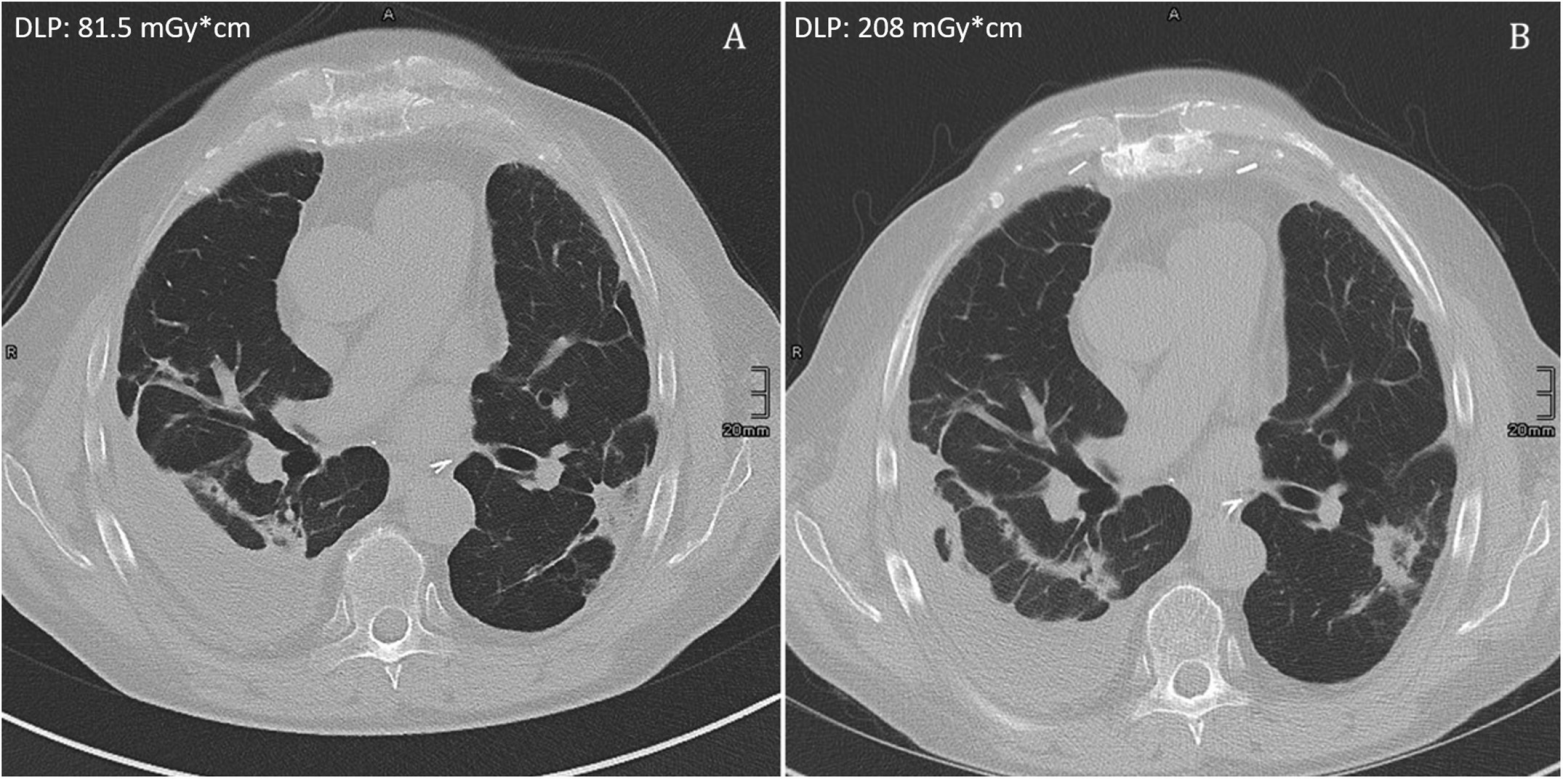
Example images of the two protocols with corresponding DLP. Images of both protocols with corresponding dose-length product (DLP). A) 3rd generation Sn150kVp protocol: DLP = 81.5 mGy*cm. B) 2nd generation 100kVp protocol: DLP = 208 mGy*cm.

All studies were considered being of diagnostic quality. Both chest CT protocols yielded a high diagnostic image quality for the assessment of especially small anatomical structures in the lung periphery and pathological changes after lung transplantation expressed in a median Likert rating of 1 (25th-75th-percentile: Sn150kVp chest protocol 1–1; conventional chest CT protocol 1–1). Interobserver agreement was a good for ratings of image quality (ICC = 0.73). For detailed frequencies of Likert ratings per group see Table 6. Fig 2 shows example images in the lung window setting of the two different protocols with the corresponding DLP.

**Table 6:** Frequencies of Likert rating per group.

Likert rating		n (%)	
		3^rd^ generation CT	2^nd^ generation CT
1 (excellent)	R1	80 (78.4%)	88 (86.2%)
	R2	78 (76.5%)	79 (77.5%)
2 (good)	R1	21 (20.6%)	12 (11.8%)
	R2	23 (22.5%)	22 (21.5%)
3 (fair)	R1	1 (1.0%)	2 (2.0%)
	R2	1 (1.0%)	1 (1.0%)
4 (poor) & 5 (unacceptable)	R1	0 (0.0%)	0 (0.0%)
	R2	0 (0.0%)	0 (0.0%)

*Experience in reading thoracic CT: R1—9 years; R2—3 years

## Discussion

Our results show that using a 3^rd^ generation DSCT with a Sn150kVp protocol and an advanced modeled iterative reconstruction algorithm allows for an overall radiation dose reduction of 65% with preserved image quality with respect to assessment of lung structures, bronchi as well as pathological findings in patients after lung transplantation in an intra-individual comparison. Due to the significant dose reduction overall SNR and SNR measured in lung tissue, air inside trachea, vertebral body and air outside the body was significantly lower in the Sn150kVp chest CT protocol compared to the 100kV protocol using the 2^nd^ generation DSCT in the same patients. Interestingly, SNR measured in muscle tissue was significantly higher in the Sn150kVp chest CT protocol compared to the 100kV protocol and for SNR measured in the descending aorta there was a trend for higher values in the Sn150kVp protocol, which did not reach statistical significance. These are the only two variables assessed on the soft tissue convolution kernel (Br36), whereas all other variables were assessed on the lung convolution kernel (Bl57). This may also be the explanation for the observed diverging results, since effects of high kV, use of Sn filter and ADMIRE are influenced by the image reconstruction kernel used.

In the subjective analysis, all studies were considered of diagnostic quality expressed in a median Likert rating of 1 for both protocols. Inter reader agreeability was good with an ICC of 0.73. Compared to previous studies such as *Debray et al* [21] significant pathologic findings in CT after lung transplantation are less common in the current investigation. This can mainly be explained by the different patient collectives and time points as *Debray et al* only included patients within six months after lung transplantation, a timespan where early complications after lung transplantation may be more pronounced. In contrast, the mean timespan between lung transplantation and CT examination in our study was much longer (5.8±3.8 years in the Sn150kVp chest CT protocol group and 4.7±3.8 years in the 2^nd^ generation CT group), which make our results less comparable to previous referenced studies, as early postoperative changes may have regressed. Suspected fungal infection was present in one patient (1%). No case of suspected lymphoproliferative disorder or lung carcinoma was present in our collective It is noteworthy that many of the findings were very subtle and very often more than one finding occurred in one examination within the same patient (e.g. consolidation and pleural effusion or bronchial wall thickening and bronchiectasis). Indeed, after the first postoperative phase chronic lung allograft dysfunction persists to be the primary cause of death. It is mainly characterized by an obstructive spirometric pattern. As this study mainly focused on the impact of a novel low dose chest imaging protocol (Sn150kVp) in combination with advanced iterative reconstruction (ADMIRE) on radiation dose and image quality, assessed on an intraindividual basis for patients after lung transplantation, clinical and histologic evaluation of CLAD was not investigated.

Patients after lung transplantation are especially prone to radiation risks due to a combination of different factors. They need a lifelong immunosuppressive therapy, which is associated with a 60-fold higher cancer risk compared to the general population [4]. In addition, some patients receiving a lung transplantation (cystic fibrosis or pulmonal arterial hypertension patients) are quite young and the timespan for accumulating radiation damages which could potentially develop into cancer is long. These factors combined with the need for regular radiographic examinations, most importantly CT scans, underlines the necessity of keeping CT radiation dose as low as possible.

One of the most efficient and most straightforward methods to reduce radiation dose is to lower the X-ray tube voltage, since the radiation dose directly relates to the square of the tube voltage [23]. However, a large part of photons that are in the lower energy spectrum do not exit the patient. Thus, these photons do not reach the detector and are not used for image formation and diagnostic purposes [9]. The built-in 0.6 mm thick Sn filter in third-generation dual-source CT systems minimizes this effect [7, 8, 15, 17] by removing most of the lower energy photons, leading to a mean photon energy of 78.7keV at 100kVp, which is significantly higher than the mean photon energy of 66.4keVat 100kVp without Sn filtering [8]. This increased mean photon energy spectrum leads to a higher dose efficiency between x-ray photon output and detector available photons in examinations without contrast agent [16].

*Haubenreisser et al* [8] and *Braun et al* [17] evaluated the effect of spectral filtration in chest CT using a 100kVp tin filtered protocol. Both studies showed substantial radiation dose reduction of approximately 90% while maintaining diagnostic image quality when compared to a 100kVp [8] or 120kVp [17] protocol respectively, without spectral shaping. *Haubenreisser et al* [8], similar to our study, found diverging results in objective image quality, with higher SNR in the aorta and lower SNR in the trachea for the Sn100kVp protocol compared to the standard protocol. In contrast to our study they did not investigate the detectability of specific pathologies. *Braun et al* [17] evaluated unenhanced chest CTs only from patients admitted for suspected pneumonia. They evaluated the diagnostic confidence for the detection of several different pathologies and found no statistically significant difference in diagnostic confidence for the detection of pneumonia, pulmonary nodules, pleural pathologies and mediastinal abnormalities. For the detection of diffuse parenchymal lung disease diagnostic confidence was significantly higher in the control group without spectral filtration. Like in our scanning protocol, both studies used ADMIRE strength level 3 for image reconstruction. However, as also addressed by the referenced authors, these (ultra-) low dose protocols (up to 90% dose reduction) may have its greatest value in a high-contrast setting, such as in the detection or in the follow up of solid lung nodules. In patients after lung transplantation early diagnosis of even discrete changes is important to initiate adequate treatment. That is why choosing a higher peak kV (Sn150kVp) made our reduction in radiation dose less pronounced (65% in our study). Choosing a 150kVp protocol, the available tube output is much higher when using a tin filter, allowing to scan even larger patients with adequate image quality. Giving the fact that our patient population is not particularly slim, the required dose level is higher to achieve diagnostic image quality. This result may show that our protocol may be a good compromise in substantial but not dramatic radiation dose reduction and high diagnostic image quality for patients during immunosuppression such as in patients after lung transplantation. Also, it has to be mentioned that a more detailed assessment of image quality was performed in the current investigation were Likert scaling also took into account assessability of small bronchi as well as pathological findings after lung transplantation such as central and peripheral ground glass opacities.

IR techniques have previously proven effectiveness in reduction of radiation exposure while maintaining image quality compared to conventionally used FBP algorithms [24, 25]. This has also been shown for the detection of pulmonary nodules, of tiny anatomic structures and lesions important for the evaluation of diffuse lung disease [3, 11–14]. In the early years of IR, these tools mainly decreased the noise in reconstructed CT images [3, 12, 13]. Newer techniques, like model-based iterative reconstruction (MBIR), use an algorithm that can accurately model the entire optical chain and consider the noise of the system, leading to a drastic reduction in radiation dose while maintaining diagnostic image quality [3]. *Choo et al* suggested that MBIR may be a powerful tool for emphysema and airway measurements in studies obtained from low dose CT [26]. Recent studies have shown that the use of spectral filtration with comparable high tube current protocols like 100kVp or 120kVp is very dose efficient while maintaining high diagnostic image quality, even more so when combined with IR [8, 14–17]. *Lell et al* found that tube currents as high as 150kVp are dose-effective in the setting of preoperative sinus surgery planning with a third-generation DSCT when combined with a tin filter (Sn150kV) and ADMIRE [19].

One major advantage of this study is the fact that we were able to compare the two examination protocols within the same patient cohort. When comparing image quality and radiation metrics many confounders, most importantly patient thickness can influence the results. By performing an intraindividual comparison of the two imaging protocols the effect of such confounders was eliminated in this study.

Several potential limitations deserve mention. First, only patients after lung transplantation have been investigated, however the results may be extrapolated to other collectives undergoing chest CTs. This study was a single center retrospective investigation and only CT examinations from one single vendor were compared. We only performed a retrospective data analysis of already performed CTs, therefore further prospective investigations are necessary to confirm our findings. Even though an intra-individual comparison was performed, due to the timespan between the two examinations (1.1±0.7 years), changes in pathological signs cannot be excluded.

In this study we changed two parameters of the scanning protocol simultaneously (FBP vs. ADMIRE; 100kVp vs. Sn150kVp), this makes it impossible to weigh which of the two parameters is responsible to which degree for the observed dose savings. However, the focus of this study was to compare two CT protocols used in clinical routine at two given time points in order to document the rapid technical progress in just a couple of years which makes scanning protocols possible that offer similar diagnostic quality to less than half of the radiation dose.

Also, the fact that FBP was used in CT examinations from earlier generations instead of using IR algorithms available at that time, such as SAFIRE, may be regarded as a limitation of this study. Advances in IR, in particular with latest IR generation, have ultimately led to an accurate image impression. This was not the case for former IR-generations that created images that appeared rather synthetic. That is why FBP remained the standard for CT in our department at second-generation DSCT and the former available SAFIRE algorithm was not used.

The contours of bones (e.g. vertebral bones) appear sharper on the 100kVp image compared to the Sn150kVp image, thus their evaluation seems to suffer from the increased image noise. However, the primary focus of this study is the evaluation of lung parenchyma. For other clinical settings different scanning protocols may be a better option.

In conclusion, the use of a 150kVp tin-filtered chest CT protocol combined with advanced modeled iterative reconstruction algorithms allows for a significant dose reduction of about 65% compared to a standard chest CT protocol using FBP, while maintaining diagnostic image quality in patients after lung transplantation. This may be to the patients’ benefit especially when repeated follow-up chest CT examinations are necessary.

## Supporting information

S1 Data (XLSX)

## Abbreviations

ADMIRE: advanced modeled iterative reconstruction
AP: anteroposterior
ASIR: adaptive statistical image reconstruction
CTDIvol: Volume CT dose index
DLP: dose-length product
DLUTX: double lung transplantation
DSCT: dual-source CT
ED: effective dose
FBP: filtered back projection
HU: Hounsfield units
IR: iterative reconstruction
IRIS: iterative reconstruction in image space
LAT: lateral
LUTX: single lung transplantation
MBIR: model-based iterative reconstruction
ROI: region of interest
Sn: tin
Sn150kVp: 150kV peak spectral filtration chest imaging protocol
SNR: Signal-to-noise ratio
